# Impact of experienced HIV stigma on health is mediated by internalized stigma and depression: results from the people living with HIV stigma index in Ontario

**DOI:** 10.1186/s12889-021-11596-w

**Published:** 2021-09-09

**Authors:** Jason M. Lo Hog Tian, James R. Watson, Francisco Ibáñez-Carrasco, Billy Tran, Janet A. Parsons, Robert G. Maunder, Kiffer G. Card, Stefan Baral, Christian Hui, Anthony R. Boni, Monisola Ajiboye, Joanne D. Lindsay, Sean B. Rourke, The Ontario HIV Stigma Index Team

**Affiliations:** 1 MAP Centre for Urban Health Solutions, Unity Health Toronto, Toronto, Canada; 2 Institute of Medical Science, University of Toronto, Toronto, Canada; 3 Dalla Lana School of Public Health, University of Toronto, Toronto, Canada; 4 Li Ka Shing Knowledge Institute, Unity Health Toronto, Toronto, Canada; 5 Department of Physical Therapy and the Rehabilitation Sciences Institute, University of Toronto, Toronto, Canada; 6 Sinai Health System, University of Toronto, Toronto, Canada; 7 School of Public Health and Social Policy, University of Victoria, Victoria, Canada; 8 Faculty of Health Sciences, Simon Frasier University, Burnaby, Canada; 9 Department of Epidemiology, Johns Hopkins School of Public Health, Baltimore, USA; 10 Canadian HIV Stigma Index Steering Committee, Toronto, Canada; 11 Ontario Positive Asians, Toronto, Canada; 12 Ryerson University, Toronto, Canada; 13 International Community of Women Living with HIV, London, UK; 14 Department of Psychiatry, University of Toronto, Toronto, Canada

**Keywords:** HIV, Stigma, Depression, Self-rated health, Regression, Mediation

## Abstract

**Background:**

Experiences of HIV stigma remain prevalent across Canada, causing significant stress and negatively affecting the health and wellbeing of people living with HIV. While studies have consistently demonstrated that stigma negatively impacts health, there has been limited research on the mechanisms behind these effects. This study aims to identify which dimensions of stigma have significant relationships with self-rated health and examine the mechanisms by which those types of stigma impact self-rated health.

**Methods:**

We recruited 724 participants to complete the People Living with HIV Stigma Index in Ontario, designed by people living with HIV to measure nuanced changes in stigma and discrimination. The present study utilizes data from externally validated measures of stigma and health risks that were included in the survey. First, we conducted multiple regression analyses to examine which variables had a significant impact on self-rated health. Results from the multiple regression guided the mediation analysis. A parallel mediation model was created with enacted stigma as the antecedent, internalized stigma and depression as the mediators, and self-rated health as the outcome.

**Results:**

In the multiple regression analysis, internalized stigma (coefficient = −0.20, *p* < 0.01) and depression (coefficient = −0.07, *p* < 0.01) were both significant and independent predictors of health. Mediation analyses demonstrated that the relationship between enacted stigma and self-rated health is mediated in parallel by both internalized stigma [coefficient = −0.08, se = 0.03, 95% CI (−0.14, −0.02)] and depression [coefficient = −0.16, se = 0.03, 95% CI (−0.22, −0.11)].

**Conclusions:**

We developed a mediation model to explain how HIV-related stigma negatively impacts health. We found that that enacted stigma, or experiences of prejudice or discrimination, can lead to internalized stigma, or internalization of negative thoughts regarding one’s HIV status and/or increased depressive symptoms which then may lead to worse overall health. Highlighting the importance of internalized stigma and depression has the potential to shape the development of targeted intervention strategies aimed at reducing the burden of stigma and improving the health and wellbeing of people living with HIV.

## Background

Experiences of HIV stigma and discrimination remain high in Canada, causing significant stress and negatively affecting the health and wellbeing of people living with HIV [1]. Despite antiretroviral treatments (ART) that have improved the quality and quantity of life for people living with HIV [2, 3], HIV-related stigma (HIV stigma) still can create a barrier preventing individuals from accessing healthcare and promoting their own wellness [4, 5]. Rueda et al., Katz et al., and Chambers et al. have shown through meta-analyses that individuals who experience greater HIV stigma have higher viral loads, worse mental health, poorer quality of life, increased likelihood of alcohol and drug misuse, and have difficulty with treatment adherence and access to healthcare services [6–8]. Increased HIV stigma is also associated with poorer self-rated health [9, 10], however there is limited research on the mechanisms behind this effect. Understanding this interaction may help to inform the development of targeted intervention strategies aimed at overcoming or helping people manage the burden of HIV stigma on health.

While studies have demonstrated the negative impact of stigma on downstream health outcomes for years [6–8], there is limited understanding regarding which aspects of stigma are linked to health and which interpersonal, psychological, or external factors may moderate or buffer these impacts [11]. The HIV Stigma Framework was designed to address these issues by breaking down stigma into three distinct dimensions and examining how they may have different impacts on an individual’s health and wellness [11–13]. These dimensions include “enacted stigma” which refers to experiences of discrimination, prejudice, or stereotyping, “internalized stigma” which involves the endorsement of negative thoughts and beliefs surrounding HIV and applying them to the self, and “anticipated stigma” or the expectation that other people will treat you negatively in the future because of your HIV status [11–13]. Each dimension has been associated with various different health outcomes, with enacted stigma often being linked with physical health and heightened stress response [11, 14, 15], internalized stigma being associated with mental health, poor affective and cognitive functioning, lack of psychological resources, and lower treatment adherence [11, 13, 16, 17], and anticipated stigma being linked with HIV non-disclosure and increased incidence of having a chronic illness comorbidity [11, 15, 18]. The conceptualization of internalized stigma as closely linked to, or in some cases synonymous with cognitive and psychological difficulties (e.g. depression) has driven the development of intervention strategies for HIV stigma so far, however these interventions have shown lackluster results [19, 20]. A secondary function of this study is to examine whether internalized stigma and depression have independent and separate impacts on health.

The HIV Stigma Framework has helped to increase our understanding of stigma and health, however the impact of stigma on an individual’s self-rated health has gone largely uninvestigated. Incorporating self-rated health into the framework facilitates exploration of how living with HIV may impact an individual’s self-concept of health and how this interaction affects their real-life health outcomes. Focusing on self-reported overall health is especially relevant now that increased access to ART has resulted in a shift in focus for HIV research from survival rates to how people living with HIV can thrive.

Self-rated health has been measured in large scale epidemiological studies for decades due to its simplicity and robust ability to predict key health outcomes such as mortality, disability, objective health status, and use of healthcare services [21–24]. It is most commonly assessed using one question that elicits a rating of a participant’s general perception of their health on a poor to excellent response scale [25]. This makes the use of a subjective global health question an extremely efficient way to glean information about an individual’s health in a clinical or research setting. The predictive ability is generally high across genders, ethnic groups, socioeconomic statuses, and education levels which makes it an ideal outcome measure for the diverse HIV population [26–32].

While objective health evaluation using signs, symptoms, and laboratory tests are the most common ways to quantify disease and their effects on health, subjective self-rated health is beneficial as it can account for the complex interaction of biological, psychological, and social implications of illness, and how these impact an individual’s wellbeing [33]. The few studies that examine the relationship between HIV stigma and self-rated health show that greater stigma is associated with poorer self-rated health [9, 10], however they do not explore whether the dimensions of stigma have different impacts on self-rated health or the mechanisms behind these effects.

The present study utilizes data from the Ontario implementation of the People Living with HIV Stigma Index to: (1) identify which dimensions of stigma have significant relationships with self-rated health and (2) examine the mechanisms by which stigma impacts self-rated health. To understand how stigma impacts health, we use a parallel mediation model which establishes pathways between an antecedent and an outcome through two or more mediating variables. Previous studies have demonstrated a link between experienced HIV stigma and internalized stigma as well as possible consequences for depressive symptoms [15, 34, 35]. Another study has demonstrated that depression mediates the relationship between loneliness and self-rated health [36]. Building on this knowledge and the HIV Stigma Framework, we hypothesize that enacted and internalized stigma will be significant predictors of self-rated health and that the relationship between enacted stigma and self-rated health is mediated in parallel by both internalized stigma and depression.

## Methods

The People Living with HIV Stigma Index is the world’s largest social research project developed by and for people living with HIV to measure nuanced changes in stigma and discrimination [37]. This global survey tool has been implemented in more than 100 countries and relies heavily on the Greater Involvement of People Living with HIV and AIDS (GIPA) principle [38]. We utilize data from the Ontario implementation of the HIV Stigma Index which included additional externally validated scales to measure stigma and other health risks. The present analysis primarily uses data from these externally validated scales.

Trained peer research associates (PRAs) living with HIV were hired to recruit 724 people living with HIV and administer the Ontario HIV Stigma Index. Survey participants are a cross-section of people living with HIV from several regions across Ontario and include priority populations such as gay, bisexual, and other men who have sex with men (GBMSM), African/Caribbean/Black (ACB) individuals, women, youth, Aboriginal peoples, injection drug users, and individuals from rural communities. PRAs administered the HIV Stigma Index in their respective regions through face-to-face interviews lasting approximately two hours between September 2018 and August 2019. All adult participants were considered for enrollment if they were (1) HIV-positive, (2) able to adequately communicate in English or French for the duration of the interview process, and (3) willing and able to complete the interview process and provide informed consent. The study was approved by the Research Ethics Board of St. Michael’s Hospital, Unity Health Toronto.

### Study participants

Various demographic data about each participant were collected at the beginning of the survey including age, years since HIV diagnosis, gender, sexual orientation, ethnicity, education, and employment status for use as potential covariates. For multivariable analyses, gender was dichotomized into male vs. non-male, sexual orientation into heterosexual vs. non-heterosexual, ethnicity into Caucasian vs. non-Caucasian, education into high school completion or less vs. greater than high school completion, and employment status into employed vs. not employed.

### HIV stigma

To measure HIV stigma, we used the 32-item version of the HIV Stigma Scale which measures stigma using four subscales [39, 40]. The enacted stigma subscale (formerly personalized stigma) examines personal experiences of rejection, the negative self-image subscale deals with feelings of guilt or shame around being HIV positive, the disclosure concerns subscale refers to the need to conceal information regarding one’s HIV status, and the concern with public attitudes subscale measures what a person with HIV believes other people may think of them because of their HIV status [40]. The disclosure concerns and concern with public attitudes subscales were merged to form a single anticipated stigma subscale and the negative self-image subscale was renamed to internalized stigma to match the HIV Stigma Framework [11, 41]. Participants were asked to respond to each item using a 4-point Likert scale ranging from “strongly disagree” to “strongly agree”. Factor analysis showed that all items loaded into the same dimensions as described during the development of the scale [40]. Subscale scores were calculated by taking the mean of all items in the subscale with higher scores indicating greater stigma. Internal consistency was high for subscales with enacted stigma, internalized stigma, and anticipated stigma having Cronbach’s alphas of 0.940, 0.894, and 0.901 respectively.

### Depression

We utilized the 9-item Patient Health Questionnaire (PHQ-9) to measure depression. The scale covers the domains of depression as defined in the DSM-IV (which remain the same in the DSM-V) and can provide both a provisional depression diagnosis and a grade of depression symptom severity [42]. Participants were asked “over the past 2 weeks, how often have you been bothered by any of the following problems” and presented with a list of 9 depressive symptoms to respond to on a 0–3 scale from “not at all” to “nearly every day”. Total depression score was calculated by taking the sum of all items with higher scores indicating greater depression. Scores from 0 to 9 are classified as “none” or “mild” depression and scores from 10 to 27 are classified as “moderate”, “moderately severe”, or “severe” depression [42]. Factor analysis demonstrated that items on the PHQ-9 loaded on one dimension and internal consistency was strong with a Cronbach’s alpha of 0.852.

### Self-rated health

To measure self-rated health, we used the one-item self-report health question: “In general, how would you describe your health at the moment?”. Participants were asked to rate their health on a 1–5 scale from “poor” to “excellent”. This single item measure is often included in other popular validated measures on quality of life including the World Health Organization Quality of Life (WHOQOL) assessment, SF-36, and the QOL10 and allows participants to evaluate their health and life satisfaction as it relates to their own experience [43–46].

### Statistical analyses

All statistical analyses were conducted using IBM SPSS Statistics version 24 [47]. Participants with no data for any demographic variables, depression, stigma, or self-rated health were removed from analyses (*n* = 54) leaving a final sample of *N* = 676. First, we conducted multiple regression analyses to examine which variables had a significant impact on self-rated health. Variables were entered in blocks to control for certain demographic variables and to examine whether variables of interest had a significant impact on the outcome. Demographic variables were entered first, then depression was added, followed by dimensions of stigma being added separately, and lastly all dimensions of stigma were added together. Results from the multiple regression guided the mediation analysis. The parallel mediation model was created using the PROCESS macro, a regression-based tool designed for conducting mediation, moderation, and conditional process analysis [48]. The antecedent variable was enacted stigma, the mediating variables were internalized stigma and depression, and the outcome variable was self-rated health. Demographic variables were added as covariates to control for any possible confounding effect. Mediation was deemed significant if the bootstrap 95% confidence interval associated with the indirect effect did not include zero. Analysis of the indirect effect was conducted with 5000 bootstrap samples [48]. Completely standardized indirect effects were computed to compare the effects of the mediators with each other by removing the scaling of antecedent and outcome variables.

## Results

### Study participants

Table 1 contains descriptive statistics on demographics and variables of interest. Participant age ranged from 18 to 79 years (M: 47.9, SD: 11.4) and years since HIV diagnosis ranged from 0 to 39 years (M: 15.0, SD: 9.4). Most participants identified as male (63%), gay or bisexual (53%), Caucasian (57%), had greater than high school education (59%), and was not employed (65%). Over a third of participants (38%) had moderate/severe depressive symptoms. Levels of internalized stigma were high (M: 2.11, SD: 0.69) with enacted stigma being even higher (M: 2.53, SD: 0.69) and anticipated stigma being higher still (M: 2.74, SD: 0.56). This pattern is consistent with the original study validating the stigma measure [40]. For self-rated health, 5% of participants rated their health as poor, 16% as fair, 33% as good, 30% as very good, and 17% as excellent.

**Table 1:** Participant demographics and variables of interest (*N* = 676)

Variable	N or mean	% or SD
Age (Years)	47.9	11.4
Years Since HIV Diagnosis	15.0	9.4
Gender		
Male	429	63%
Female	225	33%
Transgender/Non-binary	22	3%
Sexual Orientation		
Heterosexual	281	42%
Gay/Bisexual	361	53%
Other	34	5%
Ethnicity		
Caucasian	382	57%
Africa/Caribbean/Black	155	23%
Asian	44	7%
Indigenous	63	9%
Other	32	5%
Education		
> High School	80	12%
High School	200	30%
< High School	396	59%
Employment		
Employed	234	35%
Not Employed	442	65%
Depression		
No/Mild	418	62%
Moderate/Severe	258	38%
Stigma		
Internalized	2.11	0.69
Enacted	2.53	0.69
Anticipated	2.74	0.56
Self-Rated Health		
Poor	33	5%
Fair	105	16%
Good	225	33%
Very Good	200	30%
Excellent	113	17%

*SD* Standard deviation

### Multiple regression analyses

Table 2 shows the results of multiple regression analyses examining relationships between demographic characteristics, depression, and dimensions of stigma with self-rated health as the outcome variable. Model 1 includes all demographic characteristics and only employment status was significant with unemployed individuals endorsing worse self-rated health (coefficient = −0.43, *p* < 0.01). Model 2 added depression which was a significant predictor with participants endorsing higher depression rating their health as poorer (coefficient = −0.07, *p* < 0.01). Models 3, 4, and 5 separately added enacted, internalized, and anticipated stigma respectively. Enacted stigma was not significant (coefficient = −0.10, *p* = 0.08), internalized stigma was significant (coefficient = −0.20, *p* < 0.01), and anticipated stigma was not significant (coefficient = −0.06, *p* = 0.39). Model 6 added all dimensions of stigma together and internalized stigma was the only type of stigma that was a significant predictor of self-rated health (coefficient = −0.22, *p* < 0.01). The histogram of standardized residuals and the normal P-P plot of standardized residuals indicated that the data contained approximately normally distributed errors. The residual plot showed that the data met assumptions of homogeneity of variance and linearity. Tolerance was above 0.1 and variation inflation factor (VIF) was below 10 for all variables in the model, indicating that multicollinearity was not a concern. Since a significant bivariate test is not a prerequisite for mediation analysis [48], and based on previous research [34, 35], we decided to further examine the impact of enacted stigma on health through the two significant factors from the multiple regression: internalized stigma and depression.

**Table 2:** Multivariable regression analyses predicting self-rated health from covariates, depression, and dimensions of stigma

	Model 1		Model 2		Model 3		Model 4		Model 5		Model 6	
Predictor	*b*	*p*-value	*b*	*p*-value	*b*	*p*-value	*b*	*p*-value	*b*	*p*-value	*b*	*p*-value
Age (Years)	0.00	0.91	0.00	0.37	0.00	0.31	0.00	0.33	0.00	0.37	0.00	0.29
Years Since HIV Diagnosis	−0.01	0.36	0.00	0.41	0.00	0.46	− 0.01	0.19	0.00	0.38	−0.01	0.24
Gender (non-male)	0.06	0.54	0.16	0.09	0.16	0.08	0.17	0.07	0.16	0.08	0.16	0.08
Ethnicity (non-Caucasian)	0.09	0.29	0.09	0.27	0.10	0.22	0.10	0.20	0.10	0.22	0.09	0.27
Sexual Orientation (non-heterosexual)	0.03	0.75	0.15	0.11	0.14	0.13	0.12	0.19	0.14	0.13	0.12	0.18
Education (< high school)	−0.16	0.21	−0.06	0.59	−0.06	0.62	−0.04	0.75	−0.07	0.54	−0.01	0.90
Employment (unemployed)	−0.43	**< 0.01**	−0.20	**0.02**	−0.19	**0.02**	−0.18	**0.03**	−0.20	**0.02**	−0.17	**0.04**
Depression			−0.07	**< 0.01**	−0.07	**< 0.01**	− 0.07	**< 0.01**	− 0.07	**< 0.01**	−0.06	**< 0.01**
Enacted Stigma					−0.10	0.08	–		–		−0.08	0.29
Internalized Stigma					–		−0.20	**< 0.01**	–		− 0.22	**< 0.01**
Anticipated Stigma					–		–		−0.06	0.39	0.12	0.21
Constant	3.646	< 0.01	4.191	< 0.01	4.432	< 0.01	4.594	< 0.01	4.351	< 0.01	4.498	< 0.01

*b* unstandardized coefficients

### Mediation analysis

**Fig. 1:**
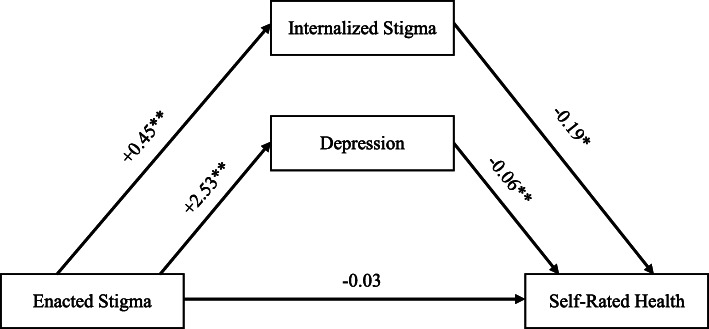
Path diagram of parallel mediation model with unstandardized beta coefficients. Covariates included: age, years since HIV diagnosis, gender, ethnicity, sexual orientation, education, employment

Using mediation analysis, we found that there is a relationship between enacted stigma and self-rated health that is mediated in parallel by both internalized stigma and depression (see Fig. 1 and Table 3 for the path diagram and details of the mediation model). In this model, the indirect effect of enacted stigma on self-rated health through internalized stigma [coefficient = −0.08, se = 0.03, 95% CI (−0.14, −0.03)] and depression [coefficient = −0.16, se = 0.03, 95% CI (−0.22, −0.11)] as mediators was significant. Overall, the final mediation model predicted 21% of the variance in self-rated health (R^2^ = 0.21, *p* < 0.01). The total effect of enacted stigma on self-rated health was also significant [coefficient = −0.27, se = 0.06, 95% CI (−0.39, −0.16)]. The steps in the first mediation pathway i.e. from enacted stigma to internalized stigma [coefficient = 0.45, se = 0.03, 95% CI (0.38, 0.51)] and from internalized stigma to self-rated health [coefficient = −0.19, se = 0.07, 95% CI (−0.32, −0.06)] were statistically significant. The steps in the second mediation pathway i.e. from enacted stigma to depression [coefficient = 2.53, se = 0.32, 95% CI (1.90, 3.16)] and from depression to self-rated health [coefficient = −0.06, se = 0.01, 95% CI (−0.08, −0.05)] were also significant. Standardized indirect effects indicated that the effect of enacted stigma through depression [β = −0.10, se = 0.02, 95% CI (−0.14, −0.07)] had a greater impact on health than through internalized stigma [β = −0.05, se = 0.02, 95% CI (−0.09, −0.02)]. The direct effect of enacted stigma on self-rated health controlling for the mediators was not significant [coefficient = −0.03, se = 0.06, 95% CI (−0.15, 0.09)].

**Table 3:** Regression coefficients, standard errors, and model summary information for parallel mediator model with enacted stigma as the antecedent, internalized stigma and depression as the mediators, and self-rated health as the outcome

	Consequent								
	Internalized Stigma			Depression			Self-Rated Health		
Antecedent	Coefficient (se)	*p*-value	(95% CI)	Coefficient (se)	*p*-value	(95% CI)	Coefficient (se)	*p*-value	(95% CI)
Enacted Stigma	0.45 (0.03)	< 0.01	(0.38, 0.51)	2.53 (0.32)	< 0.01	(1.90, 3.16)	−0.03 (0.06)	0.63	(−0.15, 0.09)
Internalized Stigma	–	–	–	–	–	–	−0.19 (0.07)	< 0.01	(−0.32, − 0.06)
Depression	–	–	–	–	–	–	− 0.06 (0.01)	< 0.01	(− 0.08, − 0.05)
Indirect Effect (Int Stig)	–	–	–	–	–	–	− 0.08 (0.03)	–	(− 0.14, − 0.03)
Indirect Effect (Dep)	–	–	–	–	–	–	− 0.16 (0.03)	–	(− 0.22, − 0.11)
Constant	1.13 (0.15)	< 0.01	(0.83, 1.43)	0.88 (1.44)	0.54	(−1.95, 3.71)	4.64 (0.26)	< 0.01	(4.13, 5.15)
	R2 = 0.27			R^2^ = 0.16			R^2^ = 0.21		
	F(8, 667) = 31.62, *p* < 0.01			F(8, 667) = 16.10, *p* < 0.01			F(10, 665) = 17.91, *p* < 0.01		

*Note* Pathways shown are unstandardized beta coefficients; Int Stig = Internalized Stigma, Dep = Depression

Covariates included: age, years since HIV diagnosis, gender, sexual orientation, ethnicity, education, employment

## Discussion

This study aims to identify which dimensions of stigma are most connected to an individual’s self-rated health and the mechanisms by which those dimensions impact the health and wellbeing of people living with HIV. Out of the types of stigma, feelings of guilt or shame around living with HIV (internalized stigma) had a significant impact on health. An individual’s mood state (depression) and employment status also had significant impact on health. While enacted stigma was not a significant predictor of health in the multiple regression analysis like we hypothesized, our mediation model demonstrated that there is a relationship between enacted stigma and self-rated health that is mediated in parallel by internalized stigma and depression.

Anticipated stigma did not show any significant relationship with self-rated health despite being highly endorsed. Anticipated stigma may manifest itself more as an external fear that influences social interactions and impacts disclosure behavior or engagement with the healthcare system rather than an individual’s self-rated health [11–13, 15]. This may also indicate that the strength of the predictors which are already significant may overshadow any potential small effect of anticipated stigma.

These findings combine useful conceptualizations of stigma mechanisms from the HIV Stigma Framework [11, 13] with research demonstrating the ability of self-rated health to translate into important real-life health outcomes [21–24]. By incorporating self-rated health into the framework, we begin to understand how stigma impacts overall health and wellbeing. We found that experiences of stigma can lead to increased internalized stigma (a social process which manifests as a chronic stressor that develops over time) and depression (a low mood state that is evaluated over a shorter period of weeks or months). These are two possible pathways through which enacted stigma impacts health and wellbeing.

Our model suggests that addressing enacted stigma would improve self-rated health (possibly by reducing feelings of guilt, shame, and depression), however a proven intervention has yet to have been developed [20, 49, 50]. Reducing instances of enacted stigma would likely require a combination of individual, community, and societal changes coupled with regional, national, and international policy changes and implementation strategies [20, 51]. This is the goal we are all striving toward, but the scope of the work to be done may discourage individual people living with HIV who are struggling with experiences of discrimination, prejudice, or stereotyping on a day-to-day basis. Conceptually, it is difficult for an individual to reduce their own enacted stigma. Our mediation model suggests that addressing internalized stigma and depression may be an effective complement to reduce the impact of stigma on health if we cannot reduce enacted stigma itself.

Internalized stigma has historically been linked with adverse psychological and mental health outcomes, especially depression [11, 13]. However, conceptualizing internalized stigma as so closely related to depression may discount the full impact it has on health and wellbeing and place the onus on the person feeling the stigma to overcome it. Thus far, intervention strategies for internalized stigma have focused on mental health-related intervention including psycho-education, increasing self-esteem, and personal empowerment, however these have shown suboptimal results [19, 20]. Despite this conceptual conflation, our multiple regression model shows that both internalized stigma and depression have significant impacts on self-rated health, even when included together in the model. This led us to assess them both as mediators in our mediation model to determine if they provided separate pathways for the effect of enacted stigma on self-rated health. Our parallel mediation model indeed demonstrated that, while they may be related, internalized stigma is a distinct factor from depression. This suggests that there may be different factors that impact internalized stigma and depression even though both may be consequences of enacted stigma and contribute to worse overall health. Our findings shift the conceptualization of internalized stigma away from being synonymous with depression and invite further research to investigate internalized stigma through a social and structural lens in addition to understanding important psychological contributors.

Given the persistently high level of stigma among people living with HIV in Ontario, identifying internalized stigma and depression as major contributing factors could be key in designing interventions for stigma reduction. There are already many proven effective interventions for depression that could reduce the negative impact of experiencing stigma on health [52–54]. Current internalized stigma interventions mostly focus on individual-level factors, however these have shown limited efficacy [19, 20]. Future research could include the development and evaluation of evidence-based interventions designed to reduce levels of internalized stigma. This includes expanding beyond psychology-based interventions and incorporating social and structural forces that play a large role in internalized stigma [19]. Developing internal and external resources such as resiliency, self-efficacy, and social support may also be helpful in overcoming the burden of stigma and improve the overall health and wellbeing of people living with HIV.

The findings from this study should be interpreted in light of some limitations. Mediation is ultimately a causal explanation which assumes that the mediator is located causally between the antecedent and the outcome [48]. The cross-sectional nature of this study means that we cannot make conclusions about the directionality and causal nature of these relationships, however this does not preclude the use of mediation and we conduct these analyses based on previous research and logical relationships between variables. Future longitudinal research must be done to examine whether stigma impacts health in the direction conceptualized here. The study is also limited by how well the sample represents all people living with HIV. While the study team made a concerted effort at recruiting participants from various regions, socioeconomic statuses, and ethnicities to adequately represent people living with HIV in Canada, there will inevitably be gaps, leaving a subset of people living with HIV that are unrepresented in the data. We also do not account for other types of non-HIV stigma such as stigma associated with gender, sexual orientation, and race/ethnicity which have been associated with key health outcomes including depression and low treatment adherence [55–57]. These are sure to be present in such a diverse sample and future research must examine how these intersectional stigmas interact with each other to influence health and wellbeing of people living with HIV. Lastly, while self-rated health has been shown to predict real-life health outcomes [21–24], we did not directly measure these outcomes, so the connection in this study is purely implied. Future work must be done to explore whether the impact of stigma through these pathways can be extended to real-life health outcomes that have been linked with self-rated health in the literature.

## Conclusion

This study brings together the HIV Stigma Framework as a conceptual model with study on the utility of a single-item self-rated global health question to predict key health outcomes. Our findings shed light on the types of stigmas that affect an individual’s health, with internalized stigma having a significant impact. We developed a mediation model to explain how stigma impacts health and found that enacted stigma can lead to internalization of negative thoughts regarding one’s HIV status (internalized stigma) and/or increased depressive symptoms which then may lead to worse overall health. Highlighting the importance of internalized stigma and depression has the potential to shape the development of targeted intervention strategies aimed at reducing the burden and mitigating the effects of stigma and improve the health and wellbeing of people living with HIV.

## Data Availability

The datasets used and/or analysed during the current study are available from the corresponding author on reasonable request.

## Abbreviations

HIV: Human Immunodeficiency Virus
ART: Antiretroviral Therapy
PRA: Peer Research Associate

## References

[1] Public Health Agency of Canada. Accelerating our response: Government of Canada five-year action plan on sexually transmitted and blood-borne infections [Internet]. 2019 [cited 2020 June 24]. Available from: https://www.canada.ca/en/public-health/services/reports-publications/accelerating-our-response-five-year-action-plan-sexually-transmitted-blood-borne-infections.html#a0-2. 10.14745/ccdr.v45i12a04 PMC7041658 32167085

[2] Hogg RS, Heath KV, Yip B, Craib KJ, O'Shaughnessy MV, Schechter MT, Montaner JS (1998). Improved survival among HIV-infected individuals following initiation of antiretroviral therapy. J Am Med Assoc.

[3] Jensen-Fangel S, Pedersen L, Larsen CS, Tauris P, Møller A, Sørensen HT (2004). Low mortality in HIV-infected patients starting highly active antiretroviral therapy: a comparison with the general population. AIDS..

[4] Sweeney SM, Vanable PA (2016). The association of HIV-related stigma to HIV medication adherence: a systematic review and synthesis of the literature. AIDS Behav.

[5] Sayles JN, Wong MD, Kinsler JJ, Martins D, Cunningham WE (2009). The association of stigma with self-reported access to medical care and antiretroviral therapy adherence in persons living with HIV/AIDS. J Gen Intern Med.

[6] Rueda S, Mitra S, Chen S, Gogolishvili D, Globerman J, Chambers L, Wilson M, Logie CH, Shi Q, Morassaei S, Rourke SB (2016). Examining the associations between HIV-related stigma and health outcomes in people living with HIV/AIDS: a series of meta-analyses. BMJ Open.

[7] Katz IT, Ryu AE, Onuegbu AG, Psaros C, Weiser SD, Bangsberg DR, Tsai AC (2013). Impact of HIV-related stigma on treatment adherence: systematic review and meta-synthesis. J Int AIDS Soc.

[8] Chambers LA, Rueda S, Baker DN, Wilson MG, Deutsch R, Raeifar E, Rourke SB, Team TSR (2015). Stigma, HIV and health: a qualitative synthesis. BMC Public Health.

[9] Emlet CA, Brennan DJ, Brennenstuhl S, Rueda S, Hart TA, Rourke SB, the OHTN Cohort Study Team (2013). Protective and risk factors associated with stigma in a population of older adults living with HIV in Ontario, Canada. AIDS Care.

[10] Hutton VE, Misajon R, Collins FE (2013). Subjective wellbeing and ‘felt’stigma when living with HIV. Qual Life Res.

[11] Earnshaw VA, Smith LR, Chaudoir SR, Amico KR, Copenhaver MM (2013). HIV stigma mechanisms and well-being among PLWH: a test of the HIV stigma framework. AIDS Behav.

[12] Earnshaw VA, Chaudoir SR (2009). From conceptualizing to measuring HIV stigma: a review of HIV stigma mechanism measures. AIDS Behav.

[13] Turan B, Hatcher AM, Weiser SD, Johnson MO, Rice WS, Turan JM (2017). Framing mechanisms linking HIV-related stigma, adherence to treatment, and health outcomes. Am J Public Health.

[14] Pascoe EA, Smart RL (2009). Perceived discrimination and health: a meta-analytic review. Psychol Bull.

[15] Turan B, Budhwani H, Fazeli PL, Browning WR, Raper JL, Mugavero MJ, Turan JM (2017). How does stigma affect people living with HIV? The mediating roles of internalized and anticipated HIV stigma in the effects of perceived community stigma on health and psychosocial outcomes. AIDS Behav.

[16] Blake Helms C, Turan JM, Atkins G, Kempf MC, Clay OJ, Raper JL, Mugavero MJ, Turan B (2017). Interpersonal mechanisms contributing to the association between HIV-related internalized stigma and medication adherence. AIDS Behav.

[17] Christopoulos KA, Neilands TB, Hartogensis W, Geng EH, Sauceda J, Mugavero MJ, Crane HM, Fredericksen RJ, Moore RD, Mathews WC, Mayer KH, Chander G, Hurt CB, Johnson MO (2019). Internalized HIV stigma is associated with concurrent viremia and poor retention in a cohort of US patients in HIV care. JAIDS Journal of Acquired Immune Deficiency Syndromes.

[18] Obermeyer CM, Baijal P, Pegurri E (2011). Facilitating HIV disclosure across diverse settings: a review. Am J Public Health.

[19] Pantelic M, Sprague L, Stangl AL (2019). It’s not “all in your head”: critical knowledge gaps on internalized HIV stigma and a call for integrating social and structural conceptualizations. BMC Infect Dis.

[20] Stangl AL, Lloyd JK, Brady LM, Holland CE, Baral S (2013). A systematic review of interventions to reduce HIV-related stigma and discrimination from 2002 to 2013: how far have we come?. J Int AIDS Soc.

[21] Idler EL, Benyamini Y (1997). Self-rated health and mortality: a review of twenty-seven community studies. J Health Soc Behav.

[22] Idler EL, Kasl SV (1995). Self-ratings of health: do they also predict change in functional ability?. J Gerontol Ser B Psychol Sci Soc Sci.

[23] Ware JE, Manning WG, Wells KB, Duan N, Newhouse JP (1984). Health status and the use of outpatient mental health services. Am Psychol.

[24] Wu S, Wang R, Zhao Y, Ma X, Wu M, Yan X, He J (2013). The relationship between self-rated health and objective health status: a population-based study. BMC Public Health.

[25] Hays RD, Spritzer KL, Thompson WW, Cella D (2015). U.S. general population estimate for "excellent" to "poor" self-rated health item. J Gen Intern Med.

[26] Abdulrahim S, El Asmar K (2012). Is self-rated health a valid measure to use in social inequities and health research? Evidence from the PAPFAM women’s data in six Arab countries. Int J Equity Health.

[27] Allen CD, McNeely CA, Orme JG (2016). Self-rated health across race, ethnicity, and immigration status for US adolescents and young adults. J Adolesc Health.

[28] Burström B, Fredlund P (2001). Self rated health: is it as good a predictor of subsequent mortality among adults in lower as well as in higher social classes?. J Epidemiol Community Health.

[29] Chandola T, Jenkinson C (2000). Validating self-rated health in different ethnic groups. Ethnicity & health.

[30] Frankenberg E, Jones NR (2004). Self-rated health and mortality: does the relationship extend to a low income setting?. J Health Soc Behav.

[31] Smith PM, Glazier RH, Sibley LM (2010). The predictors of self-rated health and the relationship between self-rated health and health service needs are similar across socioeconomic groups in Canada. J Clin Epidemiol.

[32] Zajacova A, Huzurbazar S, Todd M (2017). Gender and the structure of self-rated health across the adult life span. Soc Sci Med.

[33] PRBd SJ, Szwarcwald CL, EAd C (2011). Self-rated health by HIV-infected individuals undergoing antiretroviral therapy in Brazil. Cadernos de Saude Publica.

[34] Crockett KB, Kalichman SC, Kalichman MO, Cruess DG, Katner HP (2019). Experiences of HIV-related discrimination and consequences for internalised stigma, depression and alcohol use. Psychol Health.

[35] Fazeli PL, Turan B (2019). Experience sampling method versus questionnaire measurement of HIV stigma: psychosocial predictors of response discrepancies and associations with HIV outcomes. Stigma Health.

[36] Marziali ME, Armstrong HL, Closson K, McLinden T, Wang L, Barath J, et al. Loneliness and self-rated physical health among gay, bisexual and other men who have sex with men in Vancouver, Canada. J Epidemiol Community Health. 2020:jech-2019-213566. 10.1136/jech-2019-213566. 10.1136/jech-2019-213566 PMC7527030 32269083

[37] Global Network of People Living with HIV (GNP+). The People Living with HIV Stigma Index 2017.

[38] UNAIDS. The greater involvement of people living with HIV (GIPA): UNAIDS policy brief (2007). Joint United Nations Programme on HIV/AIDS Geneva, Switzerland; 2007.

[39] Berger BE, Ferrans CE, Lashley FR (2001). Measuring stigma in people with HIV: psychometric assessment of the HIV stigma scale. Res Nurs Health.

[40] Bunn JY, Solomon SE, Miller C, Forehand R (2007). Measurement of stigma in people with HIV: a reexamination of the HIV stigma scale. AIDS Educ Prev.

[41] Rueda S, Gibson K, Rourke SB, Bekele T, Gardner S, Cairney J (2012). Mastery moderates the negative effect of stigma on depressive symptoms in people living with HIV. AIDS Behav.

[42] Kroenke K, Spitzer RL (2002). The PHQ-9: a new depression diagnostic and severity measure. Psychiatr Ann.

[43] The WHOQOL Group (1998). The World Health Organization quality of life assessment (WHOQOL): development and general psychometric properties. Soc Sci Med.

[44] The WHOQOL Group (1998). Development of the World Health Organization WHOQOL-BREF quality of life assessment. Psychol Med.

[45] Hays RD, Sherbourne CD, Mazel RM (1993). The rand 36-item health survey 1.0. Health Econ.

[46] Muller AE, Skurtveit S, Clausen T (2016). Validating the generic quality of life tool “QOL10” in a substance use disorder treatment cohort exposes a unique social construct. BMC Med Res Methodol.

[47] IBM Corp (2016). IBM SPSS statistics for windows, version 24.0.

[48] Hayes AF. Introduction to mediation, moderation, and conditional process analysis: a regression-based approach: Guilford publications; 2017.

[49] Brown L, Macintyre K, Trujillo L (2003). Interventions to reduce HIV/AIDS stigma: what have we learned?. AIDS Educ Prev.

[50] Sengupta S, Banks B, Jonas D, Miles MS, Smith GC (2011). HIV interventions to reduce HIV/AIDS stigma: a systematic review. AIDS Behav.

[51] Grossman CI, Stangl AL (2013). Global action to reduce HIV stigma and discrimination. J Int AIDS Soc.

[52] Himelhoch S, Medoff DR, Oyeniyi G (2007). Efficacy of group psychotherapy to reduce depressive symptoms among HIV-infected individuals: a systematic review and meta-analysis. AIDS Patient Care STDs.

[53] Olatunji BO, Mimiaga MJ, O Cleirigh C, Safren SA. (2006). A review of treatment studies of depression in HIV. Topics HIV Med.

[54] Sherr L, Clucas C, Harding R, Sibley E, Catalan J (2011). HIV and depression–a systematic review of interventions. Psychol Health Med.

[55] Bogart LM, Wagner GJ, Galvan FH, Klein DJ (2010). Longitudinal relationships between antiretroviral treatment adherence and discrimination due to HIV-serostatus, race, and sexual orientation among African–American men with HIV. Ann Behav Med.

[56] Earnshaw VA, Bogart LM, Dovidio JF, Williams DR (2013). Stigma and racial/ethnic HIV disparities: moving toward resilience. Am Psychol.

[57] Logie C, James L, Tharao W, Loutfy M (2013). Associations between HIV-related stigma, racial discrimination, gender discrimination, and depression among HIV-positive African, Caribbean, and black women in Ontario, Canada. AIDS Patient Care STDs.

